# Next generation of non-contact and wireless vital sign monitoring technology in the neonatal intensive care unit: a systematic review

**DOI:** 10.1038/s41390-025-04469-0

**Published:** 2025-11-12

**Authors:** Eva Senechal, Alyssa Maximov, Emily Jeanne, Daniel Radeschi, Vivian Azevedo, Amanda Gross, Wissam Shalish, Robert E. Kearney, Guilherme Sant’Anna

**Affiliations:** 1 Department of Experimental Medicine, McGill University, Montreal, QC Canada; 2 Research Institute, McGill University Health Center, Montreal, QC Canada; 3 Department of Biomedical Engineering, McGill University, Montreal, QC Canada; 4 Division of Neonatology, Montreal Children’s Hospital, Montreal, QC Canada

## Abstract

**Background:**

Current vital sign monitoring uses skin sensors connected to monitors via wires. Emerging technologies include non-contact and wireless wearable systems. This systematic review aims to determine the current stage of development of these technologies and the prospect for clinical translation.

**Methods:**

A search on Medline, Embase, Cochrane, Scopus, and Engineering Village was conducted for studies published between January 2014 and August 2024. Two reviewers independently screened articles and extracted data on technology, signals and feasibility, safety, and accuracy outcomes. Risk of bias was assessed using the QUADAS-2; quantitative and qualitative analyses were conducted.

**Results:**

Sixty observational studies were included: 43 (72%) non-contact and 17 (28%) wireless sensors. All used a reference sensor, with a median sample of 10 patients (IQR: 6–29); and median participant characteristics were moderately preterm infants around 34 weeks of age (IQR: 31–35). Studies typically monitored a single vital sign using offline data processing with good accuracy. Risk of bias and applicability concerns were driven by small samples, unclear participant selection, and limited reporting.

**Conclusion:**

Next generation non-contact and wireless wearable technologies have the potential to enhance neonatal vital sign monitoring, but research addressing limitations and exploring feasibility and safety are needed. Standardized reporting frameworks and greater transparency are necessary for comparisons across studies.

**Impact:**

- Most emerging technologies are non-contact technologies monitoring one vital sign, usually respiratory rate (RR), and a minority were wireless wearable sensors monitoring more than one vital sign usually heart rate and RR and showed good accuracy.
- Studies had small sample sizes, short recording durations, and exclusively focused on accuracy, and often missing important information about participants and study methodology.
- Wireless and non-contact technologies show promise, and this review provides recommendations to improve study design, extend recording durations, and enhance transparency in reporting and participant selection.

## Introduction

Over 30,000 infants are born each day and 10–11% require specialized care, often in the Neonatal Intensive Care Unit (NICU).^1,2^ These infants receive around the clock care, including continuous monitoring. This involves expensive specialized medical equipment, like the wired bedside monitors, which are often inaccessible in low-resource environments.

Continuous vital signs monitoring is an essential part of patient care in the NICU. Absolute values and trends in heart rate (HR), respiratory rate (RR), oxygen saturation (SpO_2_), and temperature are used for clinical assessment and may be early indicators of clinical deterioration, informing management decisions.^3^ However, current methods to acquire these signals are challenging to patients, parents, and health care professionals as vital signs are usually obtained by the use of skin sensors connected to bedside monitors via wires; HR and RR by using adhesive electrodes, temperature via a probe secured to the skin with an adhesive, and SpO_2_ by securing an oximeter probe on the extremities, with soft adhesive band or strap.^3^ Blood pressure (BP), although not continuously monitored, is routinely checked using a BP cuff, and in rare cases using invasive arterial sampling.

Therefore, the standard of care bedside monitoring has some limitations. First, neonates, especially those born prematurely, have fragile skin susceptible to injury, and adhesives may cause tearing, irritation, and/or pain, increasing patient discomfort and the risks of infection.^2^ Additionally, the oximeter sensor must be tightly attached for accurate readings, leading to pressure sores or burns if not moved regularly.^4^ Second, wires often become tangled, restricting patient movement and proper positioning, and complicating the delivery of routine nursing care. Finally, numerous sensors and wires may negatively impact parents, reinforcing a perception of a highly medicalized environment, creating physical barriers for touching, holding, or engaging in kangaroo care.^5^ These factors may also contribute to parental fear and anxiety.

New research has focused on the development of a next generation of neonatal vital signs monitoring technologies. In recent years, two main solutions have been explored: non-contact monitoring and wireless wearable sensors. Non-contact methods, such as camera- and radar-based systems, monitor vital signs from a distance, without touching the patient, and therefore eliminating any risks to the skin. Wireless wearable sensors remove issues associated with wires and allow for unrestricted infant positioning, handling, and spontaneous movements. This systematic review aims to determine the current stage of development of these next-generation systems and whether non-invasive contactless and/or wireless wearable technologies are ready for clinical use in the NICU. To achieve this, the review will describe non-contact and wireless wearable vital sign monitoring technologies that have been developed and tested for neonatal care, investigate and describe how these technologies perform in terms of feasibility, safety, and accuracy, and determine the methodological quality of the studies.

## Methods

The review was designed in accordance with the PRISMA-P checklist for reporting systematic reviews.^6^ A protocol was made publicly available in Prospero registry for systematic reviews (ID: CRD42023455724).

### Search strategy

A search strategy was developed with the support of two librarians from McGill University: one from the faculty of Medicine and Health Sciences, and one from the faculty of Engineering. The following five databases were searched: Medline, Embase, Cochrane, Scopus, and Engineering Village. The search strategy was initially developed for Medline, and subsequently translated for other databases syntax structure. Keywords were broken down into three categories: device-related, vital signs, and population. The selected keywords were then used to search the electronic databases for relevant articles. The initial search strategy was conducted in August 2023 and was updated in August 2024. The full search strategy can be found in Supplementary Table S1.

### Eligibility criteria

This review only included original research on non-contact and wireless wearable vital sign monitoring devices for the NICU, published after 2014, as the topic of this review pertains to recent technological advances. Thus, all eligible articles were published between January 2014 and August 2024. No language restrictions were applied. Studies needed to have at least one NICU patient, and a non-contact or wireless wearable technology for continuous monitoring of vital signs. Vital signs included were HR, RR, temperature, SpO_2_, and BP. Studies that did not include any outcomes directly evaluating the technologies were excluded.

### Selection process

Full references were exported into Covidence literature review software (Veritas Health Innovation, Melbourne, Australia) for deduplication and screening. Additionally, relevant reviews were backward searched for additional articles. Selected articles underwent a two-step screening process by two independent reviewers (E.S., E.J.) using the pre-defined eligibility criteria. First, a title and abstract screening process was performed, followed by full text screening of the selected ones (E.S., E.J., D.R., A.G., A.M., V.A.). In both screening stages, any conflicts were discussed until a consensus was reached, or in cases where consensus could not be reached, a third reviewer was consulted.

### Data extraction

A standardized data collection form was developed, with input from a neonatologist (G.S.) and an engineer (D.R.), to extract the necessary information (Supplementary Methods S1). To assess what type of non-contact and wireless vital sign monitoring technologies have been developed, data related to the type of technology used, vital signs monitored, sensing principles and/or sensors used to obtain these signals, signal processing, and type of wireless communication were collected. To investigate the feasibility, safety, and accuracy of these new technologies, the following information was extracted: number and characteristics of included NICU patients, study recording duration, and outcome measures (i.e., Bland Altman, signal coverage, formal skin scoring), and the results. Finally, to appraise the methodological quality of the included studies, key disclosures related to funding and conflict of interest, and data exclusions were examined. Also, the Quality Assessment of Diagnostic Accuracy Studies (QUADAS) 2 checklist was applied. Data extraction was also done by two independent reviews (E.S., A.M.), and both forms appraised to reach a consensus.

### Data analysis

Both quantitative and qualitative syntheses methods were used to analyze the data. Descriptive measures of all outcomes were computed for the two different types of technologies, and values were tested for statistical significance using the Chi-square test of independence, or Fischer’s exact test in cases of values <5 for categorical outcomes, and Mann–Whitney U test for quantitative outcomes; *p* values for each statistical test are reported. All statistical analysis was completed using MATLAB (MathWorks, Natick, MA). The QUADAS 2 checklist, used to assess risk of bias, was incorporated in the data collection form. Additionally, a sub-analysis was conducted to compare results from non-contact and wireless wearable technologies. Given the diversity in device types, vital signs monitored, and metrics utilized to examine technologies, a meta-analysis, which requires comparable data across studies for meaningful aggregation, was not conducted.

Additional analysis and results presented in the Supplementary Material will be labeled using the prefix E.

The study references corresponding to the descriptive measures presented in the results is provided in Supplementary Table S2.

## Results

The search strategy yielded a total of 4814 results. After removing duplicates, 3050 articles underwent title and abstract screening, and 259 were selected for full-text screening. Ultimately, 60 articles where included (Supplementary Fig. S1).^7–66^ The majority were published in the last five years and originated from Europe (40%), Asia (32%), and North America (18%). A figure and table specifying the year of publication, title, and journal for each article included are provided in Supplementary Fig. S2 and Table S3. All studies were prospective observational and included a reference sensor for comparison. Included articles focused on NICU participants, but 13 (22%) also had some non-NICU patients, 3 included healthy term infants, and 10 included adults.^7,22,31,33,38,45,48,50,52,55^ Additionally, one study, which took place in a Medium Care Neonatal Unit, was included as the study population included extremely pre-term infants. The median number of total and NICU participants in studies was 10 (IQR: 6–29; IQR: 6–21, respectively). Thirty-two (53%) studies detailed specific inclusion and exclusion criteria or reported on the gestational age (GA) at birth. Twelve (20%) studies reported on the postmenstrual age and/or 25 (42%) included days of life at data recordings. Similarly, 17 studies (28%) reported birthweight, and/or 24 (40%) the weight at the time of the study. Many studies included low birthweight (<2500 g) and moderately preterm infants (see Table 1). In the sub-analysis, studies with wearable sensors had a significantly larger number of participants (*p* < 0.05) with no differences on GA and BW between the groups (Table 1).

**Table 1:** Participant information.

	All (*n* = 60)	Noncontact (*n* = 43)	Wearables (*n* = 17)	*p* value
Number of participants				
Median (IQR)	10 (6–29)	9 (6–19)	28 (10–70)	**0.014**
Min–Max	1–461	1–50	1–461	
NICU participants				
Median (IQR)	10 (6–21)	9 (5–19)	20 (10–39)	**0.045**
Min–Max	1–461	2–50	1–461	
Eligibility criteria				
Provided	31 (52)	19 (44)	12 (71)	0.065
Gestational age	32 (53)	22 (51)	10 (58)	0.592
Median (IQR)	32.4 (30.7–34.1)	32.5 (30.7–35.0)	31 (28.3–34.2)	0.280
Min–Max	26–38.6	26.0–38.6	28–36	
Gestational age at study	12 (20)	6 (14)	6 (35)	0.063
Median (IQR)	33.7 (31.2–35.1)	31.8 (30.9–35.8)	34 (33.3–35.0)	0.378
Min–Max	30.5–38	30.5–36.5	30.9–38	
Age at study (days)	25 (42)	18 (42)	7 (41)	0.961
Median (IQR)	23 (14–31)	19 (8–33)	23 (16.3–29.1)	1.00
Min–Max	2–50	2–50	14–36	
Birthweight	17 (28)	11 (26)	6 (35)	0.566
Median (IQR)	1600 (1460–2200)	1910 (1598–3016)	1456 (1259–1675)	**0.027**
Min–Max	1100–3100	1400–3100	1110–2200	
Weight at study	24 (40)	16 (37)	9 (53)	0.493
Median (IQR)	2247 (1700–2495)	2274 (1664–2494)	2200 (1750–2530)	1.00
Min–Max	960–3293	960–3293	1245–2600	

Legend: results are presented as *n* (%) or median (IQR).

### Non-contact and wireless vital sign monitoring technologies

A total of 43 (72%) studies were related to non-contact and 17 (28%) to wireless wearable technologies. Overall, most studies monitored only one vital sign, usually computed via offline signal processing (Table 2). A Venn Diagram showing the frequency of the specific combinations of vital signs monitored is provided in Supplementary Fig. S3. Non-contact technologies used predominantly a single-device system (67%), mostly RGB cameras (74%). Frequently monitored signs were RR (72%) and HR (51%). Vital sign generation occurred offline (79%), and rarely utilizing wireless communication. The torso was the most frequently defined region of interest, followed by the head. Details on non-contact sensing methods are provided in Table 3 and Supplementary Table S4. Wireless wearable devices also primarily utilized a single-device system (77%) often applied to the torso, and typically usings bands/elastic straps (41%). The majority computed vital signs in real-time and transmitted information using Bluetooth (76%). Most wearable devices monitored more than one vital signal (59%)—HR (71%) and RR (47%). HR was primarily achieved via electrocardiography (54%), and RR used a variety of techniques, including impedance, acoustics, and accelerometry. For SpO_2_, all wearable devices relied on photoplethysmography (PPG). Details about wearable devices sensing methods are provided in Table 4 and Supplementary Table S5.

**Table 2:** Vital sign monitoring characteristics.

	All (*n* = 60)	Noncontact (*n* = 43)	Wearables (*n* = 17)	*p* value
Signals monitored^a^				
Respiratory rate	39 (65)	31 (72)	8 (47)	
Heart rate	35 (58)	22 (51)	13 (71)	
Oxygen saturation	6 (10)	1 (2)	5 (29)	
Temperature	3 (5)	0 (0)	3 (18)	
Blood pressure	1 (2)	0 (0)	1 (6)	
Value generation				
Offline	36 (60)	34 (79)	2 (12)	*p* < 0.05
Real-time	19 (32)	4 (9)	15 (88)	
Not specified	5 (8)	5 (12)	0 (0)	
Number of signs monitored				
1	39 (65)	32 (74)	7 (41)	0.058
2	18 (30)	11 (26)	7 (41)	
3	3 (5)	0 (0)	3 (18)	

Results are presented as *n* (%).

^a^Some studies were classified into multiple categories.

**Table 3:** Non-contact technologies.

Characteristics	*n* = 43
Type of sensor^a^	
RGB camera	34 (79)
Infrared camera	12 (28)
Depth camera	5 (12)
Monochrome	5 (12)
Radar	3 (7)
Number of sensors	
Single feature device	29 (67)
Multifeatured device	7 (16)
Multiple devices	7 (16)
Information transfer^a^	
No wireless transmission	38 (88)
Impulse radio ultra-wideband	2 (5)
Wi-Fi	2 (5)
Cellular data (5 G)	1 (2)
Continuous-wave radar communication	1 (2)
Region of interest (ROI)^a^	
Torso	24 (56)
Head	22 (51)
Variable	9 (21)
Upper limbs	3 (7)
Neck	3 (7)
Lower limbs	2 (5)
No info	3 (7)

Results are presented as *n* (%).

^a^Some studies were classified into multiple categories.

**Table 4:** Wireless wearable technologies.

Characteristics	*n* = 17
Number of devices	
1	13 (77)
2	3 (18)
3	1 (6)
Placement (site of contact with body)^a^	
Torso	10 (59)
Lower limbs	6 (35)
Upper limbs	5 (29)
Head	1 (6)
Neck	0 (0)
No info	1 (6)
Method of placement^a^	
Band/strap/elastic	7 (41)
Adhesive	6 (35)
Sock	2 (12)
Vest	1 (6)
Cap	1 (6)
N/A not specified	1 (6)
Information transfer^a^	
Bluetooth	13 (76)
No wireless transmission	1 (6)
Near Field Communication	1 (6)
IEEE 802.15.4 protocol	1 (6)
Wi-Fi	1 (6)
Method not specified	1 (6)

Results are presented as *n* (%).

^a^Some studies were classified into multiple categories.

### Feasibility, safety, and accuracy of the new technologies

Feasibility was often measured as the amount of usable data or the processing times of the technologies. Assessments varied between the two technologies, reflecting their unique concerns; non-contact studies primarily focused on the amount of usable data, whereas wireless wearable studies emphasized coverage, particularly for real-time vital sign generation. Thirty-eight (63%) studies specified a planned recording duration, but only 24 (40%) reported the actual duration per infant. When provided, the median duration was 0.32 h (IQR: 0.003–1.31). Only 19 studies (32%) reported the total recording duration across all participants, with a median of 11.3 h (IQR:1.77–441). In a sub-analysis, wearable studies had longer pre-defined and total recording durations than non-contact studies. Details on recording durations are presented in Table 5.

**Table 5:** Studies design.

Recordings duration	All (*n* = 60)	Noncontact (*n* = 43)	Wearables (*n* = 17)	*p* value
Predefined	38 (63)	25 (58)	13 (76)	0.2412
Median (IQR)	0.25 (0.15–1.00)	**0.17 (0.08–0.44)**	**1 (0.88–8.00)**	**0.013**
Min–Max	0.003–48	0.003–48	0.02–24	
Average	24 (40)	19 (44)	5 (29)	0.293
Median (IQR)	0.32 (0.06–1.31)	0.30 (0.05-0.83)	1 (0.17–8)	0.354
Min–Max	0.003–24.2	0.003–5.73	0.003 - 8	
Total	19 (32)	13 (30)	6 (35)	0.704
Median (IQR)	11.3 (1.77–441)	11.3 (0.83-42.0)	342 (9.5–657)	0.147
Min–Max	0.04–750	0.04–532	1–750	

Results are presented as *n* (%), or as median (IQR) when applicable.

Safety was only examined in 1 study, which focused on the potential impact on the fragile neonatal skin. Accuracy-related outcomes were the primary focus of 58 (97%) studies (Table 6). Accuracy was always assessed by comparing device performance to a reference measurement and used the Bland-Altman analysis (Table 6). Analyses of HR and RR using this method revealed low bias and moderately acceptable 95% limits of agreement (LoA) (Supplementary Table S6). An accuracy sub-analyses between the 25 (42%) studies, including all data, and 28 (47%) studies that excluded portions of data showed similar LoA and bias. Non-contact versus wireless wearable monitoring technologies showed low bias and LoA (Supplementary Table S6).

**Table 6:** Outcomes Reported.

	All (*n* = 60)	Noncontact (*n* = 43)	Wearables (*n* = 17)	*p* value
Outcomes				
Accuracy	58 (97)	42 (98)	16 (94)	
Feasibility	16 (27)	12 (28)	4 (24)	
Safety	1 (2)	0 (0)	1 (6)	
# of outcome categories				
1	45 (75)	32 (74)	13 (76)	0.869
2	15 (25)	11 (26)	4 (24)	
Accuracy				
Compare index to reference	58 (100)	42 (100)	16 (100)	
Endpoints^a^				
Bland Altman	39 (67)	28 (65)	11 (69)	
Basic descriptive measures	20 (34)	15 (35)	5 (31)	
RMSE	21 (36)	21 (49)	0 (0)	
Correlation	21 (36)	15 (35)	6 (38)	
MAE	20 (34)	19 (44)	1 (6)	
Significance testing	11 (19)	7 (16)	4 (25)	
Feasibility^a^				
Amount of usable data	6 (38)	6 (50)	0 (0)	
Processing time	4 (25)	3 (25)	1 (25	
Coverage	3 (19)	1 (8)	2 (50)	
Signal quality	2 (13)	1 (8)	1 (25)	
Clinician feedback	2 (13)	1 (8)	0 (0)	
Effect of environmental factors	1 (6)	1 (8)	0 (0)	
ROI detection success	1 (6)	1 (8)	0 (0)	
Endpoints^a^				
Basic descriptive measures	14 (88)	10 (83)	4 (100)	
Significance testing	4 (25)	2 (16)	2 (50)	
Other	3 (19)	3 (0.25)	0 (0)	
Correlation	2 (13)	0 (0)	2 (50)	

Results are presented as *n* (%).

*RMSE* root Mean Square Error, *MAE* mean absolute error.

^a^Some studies were classified into multiple categories. Only one study assessed safety by examining the peel force required to remove the sensor and documenting visual inspection of the skin following the removal.

### Methodological quality of studies

Forty-three (72%) studies reported a funding statement, but 27 (45%) lacked a conflict-of-interest (COI) statement (Supplementary Table S7). The QUADAS-2 assessment revealed several areas of concerns regarding risk of bias and applicability. Risk of bias related to participant selection was deemed unclear in most studies (60%) due to a lack of inclusion and exclusion criteria and small sample sizes (Supplementary Fig. S4A). Uncertainty about the applicability of participant selection was also very common (55%) since many studies lacked key basic descriptors of the population, such as age and weight, leading to challenges assessing if the sample was representative of the range of NICU patients (Supplementary Fig. S4B). Eleven (33%) studies had high applicability concerns due to exclusions of large groups of NICU patients, such as those requiring respiratory support or in incubators. Furthermore, reference measurement presented an unclear risk of bias in 35 (58%) studies due to the use of impedance as the bedside RR standard (Supplementary Fig. S4C). Applicability concerns for the reference measurement were generally low (58%), as bedside standards were deemed suitable for addressing the review question. However, 9 (15%) studies had high applicability concerns, primarily when the chosen reference was neither the bedside standard nor the gold standard (Supplementary Fig. S4D). Additionally, some studies provided incomplete descriptions of reference measurements, only naming them as “standard” or “routine” monitoring, limiting the ability to determine the risk of bias.

Risk of bias of the index measurement was determined as unclear in 34 (57%) studies due to missing information about the interpretation of results for articles which derived vital signs offline, and use of short recording sessions, which could potentially skew the results (Supplementary Fig. S4E). In terms of applicability of index test, 32 (53%) studies were deemed of low concern (Supplementary Fig. S4F). However, 8 studies (13%) were deemed high risk due to stringent experimental conditions, such as specific lighting, clothing restrictions, and modifications to the patient incubator. Risk of bias of patient study flow and timing was determined as low across 42 (70%) studies (Supplementary Fig. S4G). Regarding key declarations, 22 (51%) non-contact studies and 9 (53%) wireless wearable studies lack a COI statement. The QUADAS-2 analysis exhibited similar performance in terms of risk of bias and applicability concerns between both technologies. However, studies focusing on wearable devices tended to provide slightly more detailed information regarding participant selection criteria and larger sample sizes. The most notable difference between the two technologies was in the risk of bias associated with the index tests (*p* < 0.05); non-contact studies often used very short recording durations (limited to periods when the infant was not moving or receiving care), potentially introducing a positive bias in the results (Supplementary Table S8).

A summary of the corresponding figures and tables for each objective is provided on Supplementary Table S9.

## Discussion

This systematic review highlights the potential and challenges for novel non-contact and wireless wearable vital sign monitoring technologies to be implemented in the NICU. Unfortunately, most studies included small sample sizes, lacked clear eligibility criteria, and/or excluded large portions of NICU patients, limiting generalizability.

### Non-contact and wireless vital sign monitoring technologies

The development and testing of these technologies for NICU patients is increasing, the most frequently investigated technologies were non-contact devices primarily utilizing RGB camera to derive RR from either motion change or color changes. Wireless wearables represented a smaller portion of published studies, and primarily focused on developing Bluetooth-enabled devices for monitoring of multiple vital signs, usually HR through miniaturized ECG’s and RR through a variety of novel methods such as acoustic and pressure sensors. Both technologies were in early stages of development, with studies commonly characterized by short recording durations, small sample sizes, and a focus on stable, moderately preterm infants. Studies of wearable technologies, however, tended to report slightly longer recordings and larger cohorts.

### Feasibility, safety, and accuracy

Only a minority of studies examined feasibility or safety. They featured short recording durations and excluded data recorded during non-optimal conditions (spontaneous movements or handling for care), preventing evaluations during a variety of procedures and levels of activity. Other feasibility factors, such as signal coverage or gaps, processing time, cost, battery life, and wireless connection reliability, were not systematically evaluated. Moreover, healthcare providers’ and parents’ perspectives were not explored.

Only one study addressed safety by examining the impact of a wearable device on the skin. For wearable devices, evaluations of skin injury and pain associated with adhesives removal should be incorporated. Non-contact technologies also present unique safety concerns, such as the impact of cutting holes in incubators for cameras, and the reliability of optical methods for different skin pigmentations. Privacy concerns, particularly around de-identified video data, also need attention. Additionally, broader structural concerns related to interoperability, cybersecurity, and alarm management will need to be proactively addressed as wireless and non-contact monitoring technologies move closer to clinical implementation. In the NICU, these systems will be required to operate within a complex ecosystem of existing devices like bedside monitors, ventilators, and infusion pumps. Ensuring seamless interoperability across devices from multiple manufacturers will be essential. New technologies must also avoid contributing to alarm fatigue by minimizing unnecessary or non-actionable alerts. Wireless signal interference from other devices may affect signal integrity, with potential implications for patient safety. From a cybersecurity standpoint, wireless devices, especially those using Wi-Fi, introduce new vulnerabilities that require robust encryption and secure data transmission protocols. Bluetooth-based systems, while limited to local communication, present their own challenges, including risks of device mispairing, especially in open-bay NICUs. These risks highlight the need for intuitive yet secure authentication mechanisms to ensure correct device-to-patient matching. Ultimately, the success of these technologies will depend not only on performance but also on their thoughtful and safe integration into the broader NICU infrastructure.

Accuracy was the most commonly reported outcome, typically using the Bland-Altman method, with most studies reporting low bias and good LoA. However, few studies evaluated clinical accuracy using tools such as Clarke Error Grids or Event Analysis, which provide context on the clinical relevance of the agreement. Including these metrics would enhance clinicians’ ability to interpret the practical implications for patient care.

### Methodological quality of the studies

Significant inconsistencies and gaps in the methodology and reporting practices across studies were identified, making it challenging to assess selection bias or applicability. Demographics such as GA, postmenstrual age at study, birth weight, and/or weight at study were often missing, and only a minority of investigations reported details on respiratory support or active diagnoses. Furthermore, descriptions of reference technologies were frequently inadequate, typically labeled as “standard physiological monitors”. Data collection protocols lacked clear descriptions, including pre-defined and actual recording durations and any adjustments, such as restricting recordings to non-care periods, uncovered infants, or specific body positions. Transparent data analysis, including total data collected and explanations for any exclusions, and any data processing, is also crucial. Finally, many studies lack disclosure statements on funding and potential conflicts of interest. This is especially important given the high potential for commercialization.

### Prospect for clinical application

To obtain regulatory approval from agencies such as the U.S. Food and Drug Administration or Health Canada, new monitoring devices must demonstrate strong performance across multiple domains, either through novel evidence or by establishing substantial equivalence to an existing device.^67,68^ Beyond accuracy, which is the single focus of most studies, devices intended for use in the NICU must show consistent performance with respect to signal availability, data integrity, and processing under real-world conditions.^69^ Safety considerations are critical and should include biocompatibility (for skin-contacting devices), thermal and electrical safety, and compliance with wireless emission standards.^70–72^ Moreover, usability and integration into clinical workflows must be validated with intended end-users, such as NICU allied health care providers and parents.^73^ Research should therefore extend beyond accuracy to a comprehensive evaluation of these additional aspects of device performance.

### Proposed framework

To improve consistency and quality in this field, and to address the challenges identified in this review, we propose two published initiatives aimed at enhancing both methodological rigor and reporting standards.

First, to address reporting inconsistencies, we present an expanded checklist adapted from the 2016 CONSORT extension for pilot and feasibility studies, tailored specifically for research involving novel monitoring technologies in the NICU^74^. This checklist aims to promote standardized, comprehensive reporting, thereby improving research quality, interpretability, and relevance, and supporting the development of more robust study designs (Supplementary Table S10)^72^.

Additionally, we have developed and published a study protocol for a wireless wearable monitoring system that outlines clear methodology for data collection, and includes a detailed assessment of device accuracy, safety, and feasibility.^75^

### Beyond standard vital sign monitoring

This review focused on the minimum general monitoring requirements for all patients in the NICU: HR, RR, SpO2, temperature, and BP. However, most infants in the NICU will also undergo additional monitoring tailored to their clinical status, including neurological, gastrointestinal, and advanced hemodynamic assessments. Next-generation non-contact and wireless technologies are increasingly being developed to address these needs, with novel devices targeting non-rudimentary parameters such as wireless EEG for neurological monitoring, acoustic sensors for bowel motility, wireless near-infrared spectroscopy for cerebral oxygenation, and systems to monitor phototherapy exposure.^66,76–79^ These innovations reflect a broader movement toward more comprehensive, non-invasive monitoring in the NICU, with the potential to reduce patient burden and expand the range of parameters captured in both acute and longitudinal care.

### Limitations

This review has some limitations. First, studies that included NICU patients but did not present separate outcome data were excluded, potentially leading to the exclusion of some relevant investigation. Also, the inconsistencies in reporting patient demographics or duration of recordings, using means, medians, or ranges, introduced variability in the pooled descriptive data that may affect the precision of aggregated summaries. Further, this heterogeneity prevented more detailed analysis of the characteristics of key participant subgroups, like premature or low birthweight infants. Finally, while included studies showed promising accuracy results, the potential for publication bias toward positive findings should be acknowledged. As a result, accuracy may have been overestimated, and outcomes related to feasibility or suboptimal performance underreported or omitted.

## Conclusion

Next generation non-contact and wireless wearable technologies have the potential to enhance vital sign monitoring in the NICU, but future research addressing current limitations and exploring feasibility and safety are needed. Standardized reporting frameworks and greater transparency are necessary for comparisons across studies.

## Supplementary information

Supplementary information

Supplementary information

Supplementary information

Supplementary information

Supplementary information

## Data Availability

Full set of extracted data used in the systematic review will be made available upon reasonable request.
