# Supplementary material for: Next generation of non-contact and wireless vital sign monitoring technology in the neonatal intensive care unit: a systematic review
Source: Pediatr Res. 2025 Nov 12;99(7):2490–8. doi: 10.1038/s41390-025-04469-0 (PMC13485688; doi:10.1038/s41390-025-04469-0)
Supplement: Supplementary file 1 [file 41390_2025_4469_MOESM1_ESM.pdf]

Supplementary Table S1 – Search Strategy

**Device key terms**	wearable* or noncontact or non-contact or patch* or camera or belt* or diaper* or band* or wristband* or sock or socks or jacket* or vest or vests or video* or remote sens* or wireless or non-wired or unwired
**Vital sign key terms**	HR or ECG or electrocardio* or pulse or puls*rate or plethysmograph* PPG or resp* rate* or oxygen saturation* or body temperature* or blood pressure or electric impedance or oximet* or vectorcardiograph* or ballistocardiograph* or skin temperature* or vital sign* or blood pressure
**Population key terms**	NICU or neonat* intensive care unit* or neonat* ICU

Legend: * indicates all words with this root were searched.

Supplementary Methods S1 – Data Extraction Form Items

Section 1: General Information

- Title of paper.
- Study DOI.
- Year of publication.
- Journal published in
- Author(s).
- Corresponding authors contact information
- Number of study sites.
- Continent in which study was conducted.
- Country in which study was conducted.
- Study funding source.
- Do the authors have any conflict of interest to declare?

Section 2: Study Characteristics

- Aim of the study
- Study Design
- Study location (i.e. NICU, clinic, et…)
- Inclusion Criteria
- Exclusion Criteria
- Did the study include non-NICU participants
- Total number of participants
- Total number of NICU participants
- Were any of the following groups of infants included in the study
    - Preterm infants
    - Term infants
    - Very low birthweight infants (<1500)
    - Low birthweight infants (<2500)
    - Infants on CPAP
    - Infants on NIPPV
    - Infants on HF/LF canula
    - Infants of MV
- NICU participant clinical information

	Mean/Median	Min	Max	Other
**GA**				
**PMA**				
**DoL**				
**BW**				
**CW**				

- Other relevant participant information:

Section 3: Study Methods

- Was a reference technology used?
- Name of the reference technology:
- Was there a pre-defined recording duration?

- What was pre-defined recording duration?
- Recording duration (actual)

	State group (i.e. all or subgroup)	Min recording period	Max recording period	Mean/median recording period	Variability in recordings	Total duration of recording
1						
2						
3						

- Types of outcomes reported in the study:
  - Safety
  - Feasibility
  - Accuracy
  - Clinical
  - Other

- Provide brief description of what outcomes were assessed and their endpoints

Outcome	Describe what was assessed	Endpoint
Safety		
Feasibility		
Accuracy		
Clinical		
Other		

Section 4: Study Results

- Summarize the findings for each type of outcome reported.

Outcome	Main findings
Safety	
Feasibility	
Accuracy	
Clinical	
Other	

- Select any of the following reported for feasibility:
  - Basic descriptive measures (mean, median, mode, min, max, IQR, SD, variance)
  - Bland Altman
  - Mixed Effect Model
  - Correlation
  - Variation accounted For
  - Parametric significance testing
  - Non-parametric significant testing
  - Other:
- Values for any of the feasibility outcomes selected above.
- Select any of the following reported for safety:
  - Basic descriptive measures (mean, median, mode, min, max, IQR, SD, variance)

- Bland Altman
    - Mixed Effect Model
    - Correlation
    - Variation accounted For
    - Parametric significance testing
    - Non-parametric significant testing
    - Other:
- Values for any of the safety outcomes selected above.
- Select any of the following reported for accuracy:
    - Basic descriptive measures (mean, median, mode, min, max, IQR, SD, variance)
    - Bland Altman
    - Mixed Effect Model
    - Correlation
    - Variation accounted For
    - Parametric significance testing
    - Non-parametric significant testing
    - Other:
- Values for any of the accuracy outcomes selected above.

Section 5 – Monitoring Technology Information

- Device name.
- Type of technology being tested (non-contact and/or wireless).
- For non-contact monitoring system please specify relevant equipment for non-contact recording.
- How many devices are placed on the infant in the study
- Where are wearables applied to the body OR if what is the ROI for non-contact technologies
    - Head
    - Neck
    - Torso
    - Upper limbs
    - Lower limbs
    - N/A (not specified)
- Provide specific wearable/ROI defined location.
- Were any modifications made to NICU set up/care for study
- Were vital signs computed in real-time or offline.
- Which vital signs were monitored by the device.
- Which methods were used to monitor HR.
- Which methods were used to monitor RR.
- Which methods were used to monitor SpO2.
- Which methods were used to monitor temperature.
- Which methods were used to monitor blood pressure.
- Additional information about vital signs monitored (i.e. lead configuration, averaging windows, etc…)

	**Additional Information**
**HR**	
**RR**	
**SpO2**	

**Temp**	
**Blood pressure**	
**Other**	

- Did the device monitor non-vital signs?
- What non-vital signs were monitored

Section 6 – Study Quality Assessment

- Did the results include all participants data in analysis?
  - Yes
  - No
  - Unsure
- If selected "No" please indicate explanation/justification for data exclusions provided.
- If indicated provide number of hours/participants excluded from the study
- Does the selection process of patients introduce bias? Risk:
  - High
  - Low
  - Unclear
- Please provide justification if "High" or "Unclear" selected in previous question.
- Was the study population appropriate for review question? Concern:
  - High
  - Low
  - Unclear
- Please provide justification if "High" or "Unclear" selected in previous question.
- Does the conduct or the interpretation if the index test introduce bias? Risk
  - High
  - Low
  - Unclear
- Please provide justification if "High" or "Unclear" selected in previous question.
- Does the conduct or the interpretation of the index test differ from the review question? Concern:
  - High
  - Low
  - Unclear
- Please provide justification if "High" or "Unclear" selected in previous question.
- Does the conduct or the interpretation if the reference test introduce bias? Risk
  - High
  - Low
  - Unclear
- Please provide justification if "High" or "Unclear" selected in previous question.
- Does the conduct or the interpretation of the reference test differ from the review question? Concern:
  - High
  - Low
  - Unclear
- Please provide justification if "Low" or "Unclear" selected in previous question.
- Could the flow and timing of study procedures (reference and index) introduce bias? Concern:
  - High

- Low
- Unclear

Concern for other sources of bias?

Supplementary Figure S1. Study Selection Flowchart

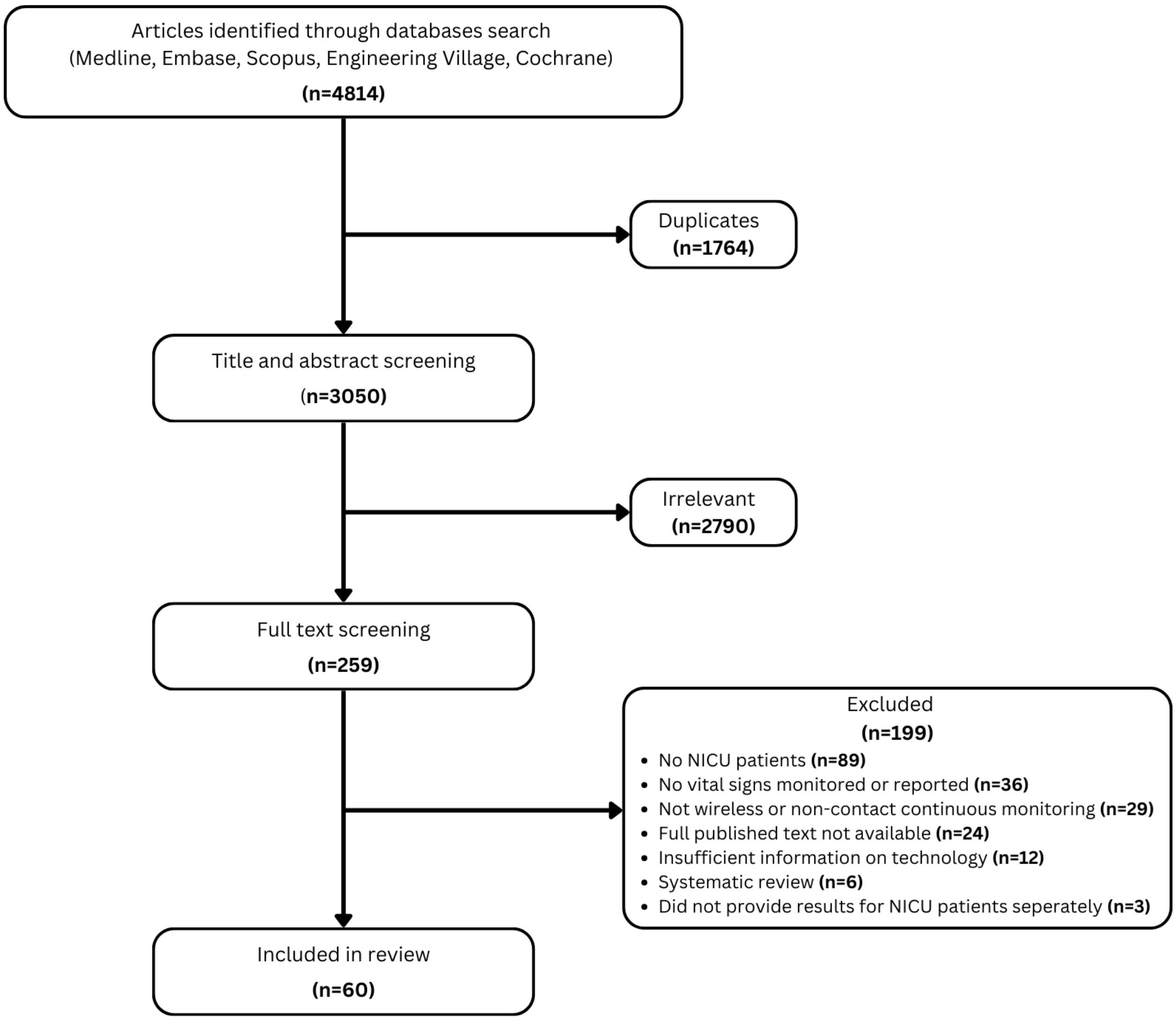

Legend: Prisma flowchart showing number of articles excluded in each step of the screening process.

Figure E2. Study Demographic Information

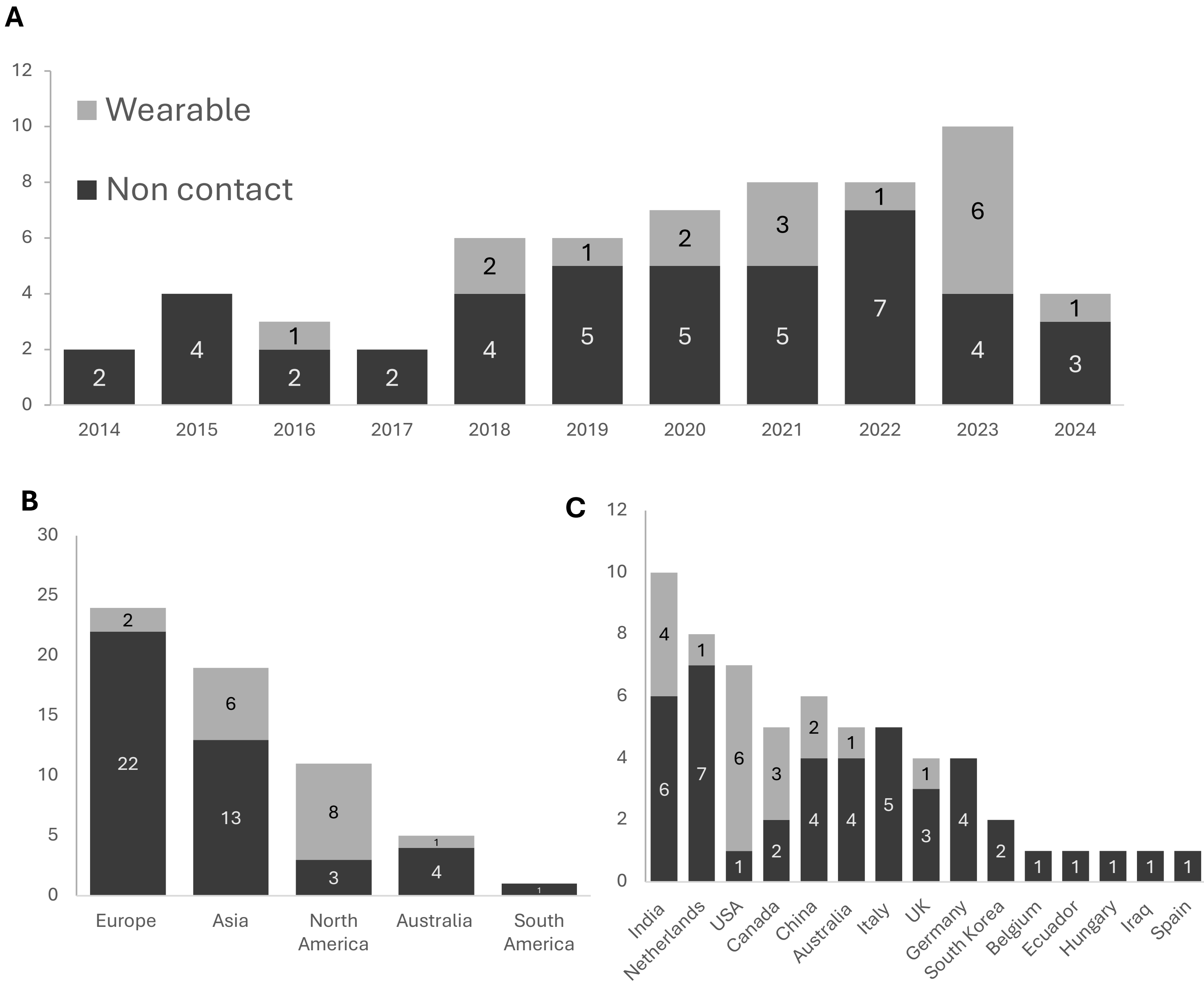

Legend: Panel A – Bar chart representing number of articles included in this review published per year. Panel B represents continents from which articles originated, and Panel C represents the country. Bars in panels A, B, and C are divided by purple, representing articles discussing wearables, and navy blue representing non-contact articles.

Supplementary Figure S3. Venn Diagram of Vital Signs Monitored

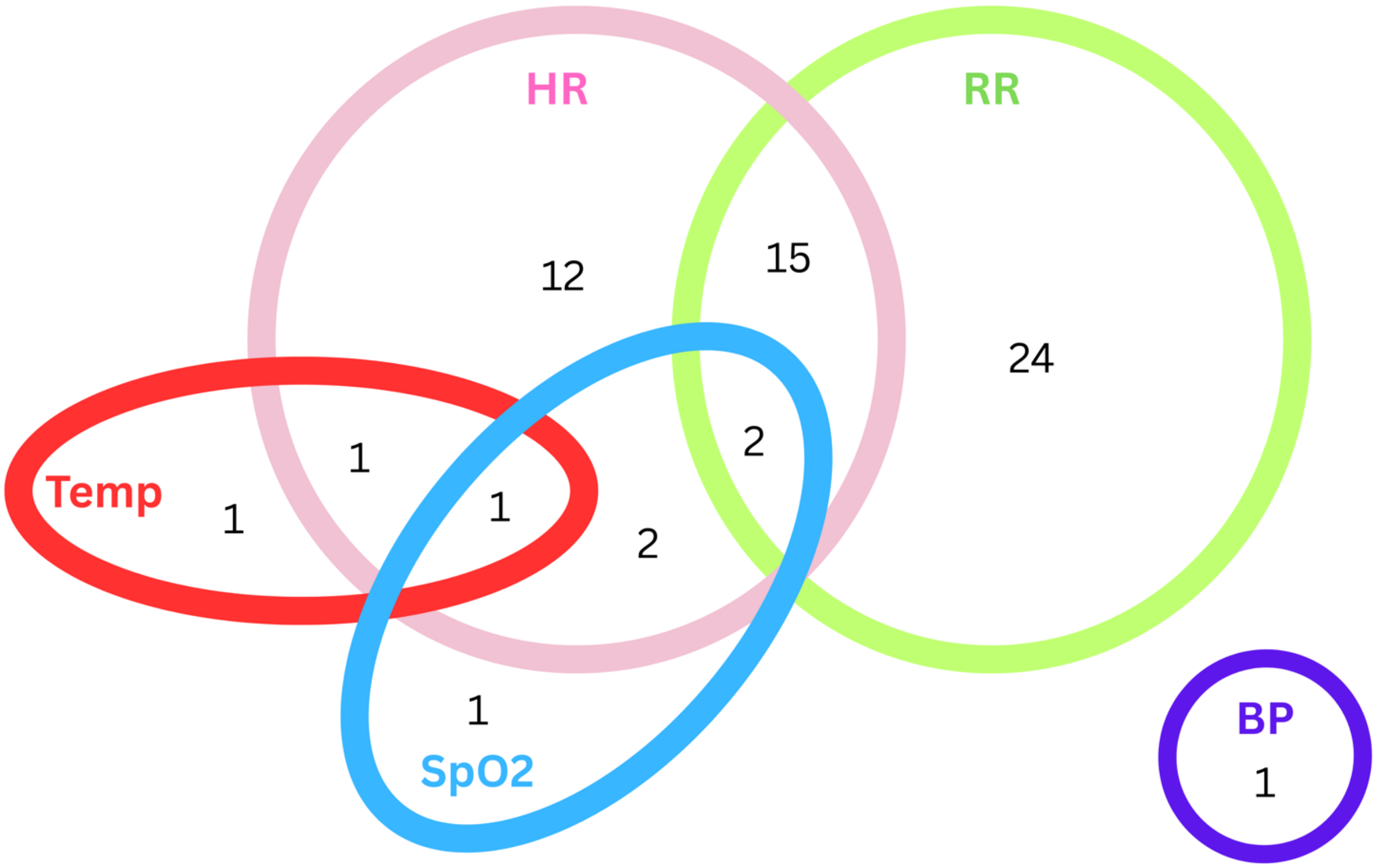

Legend: Numbers represent number of devices monitoring each combination of vital signs for included articles. Heart Rate (HR), Respiratory Rate (RR), Oxygen Saturation (SpO_2), Temperature (Temp), Blood Pressure (BP).

Supplementary Figure S4. QUADAS 2 Assessment

(A) Study Participants – Risk of Bias

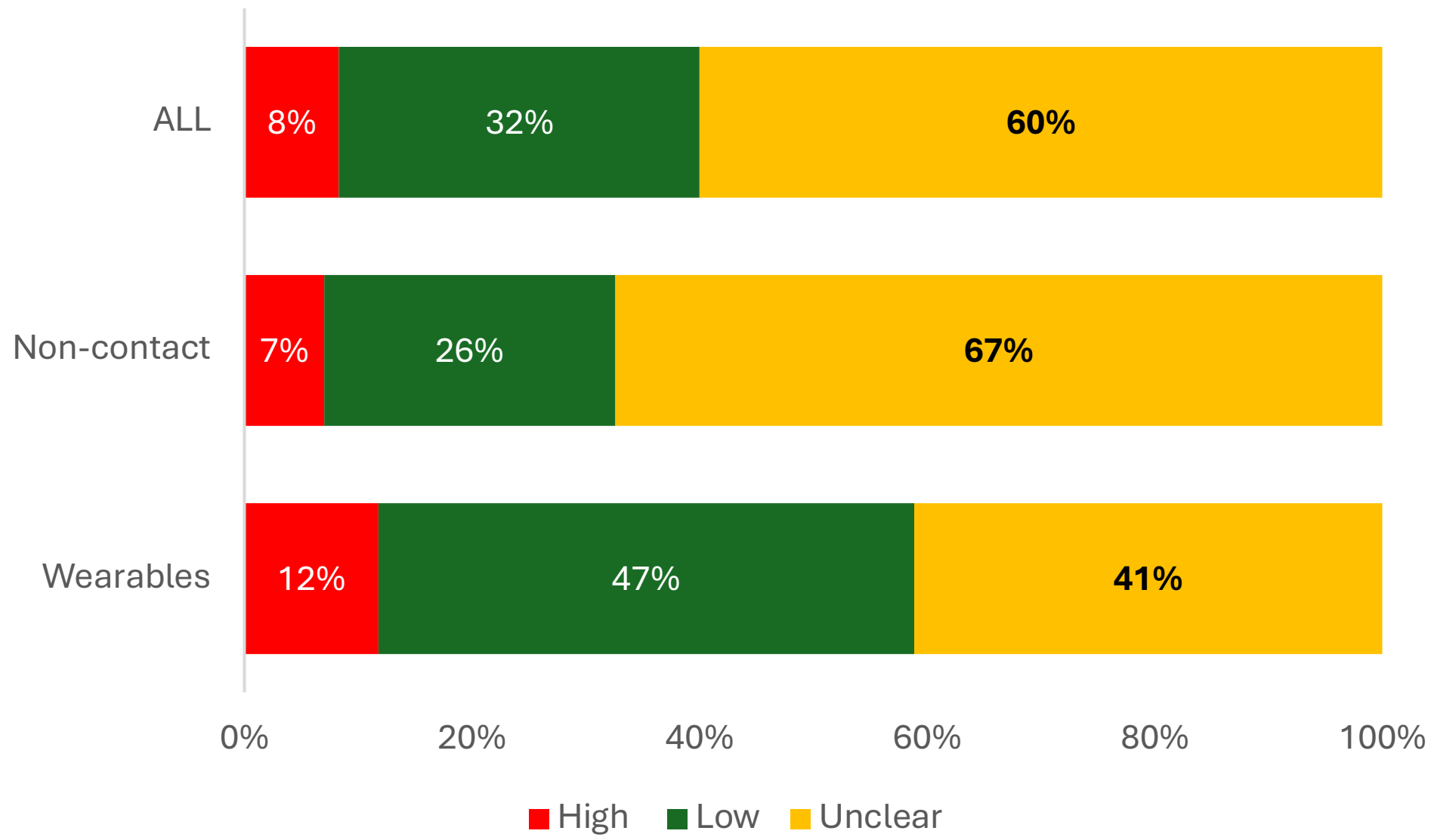

(B) Study Participants - Applicability Concerns

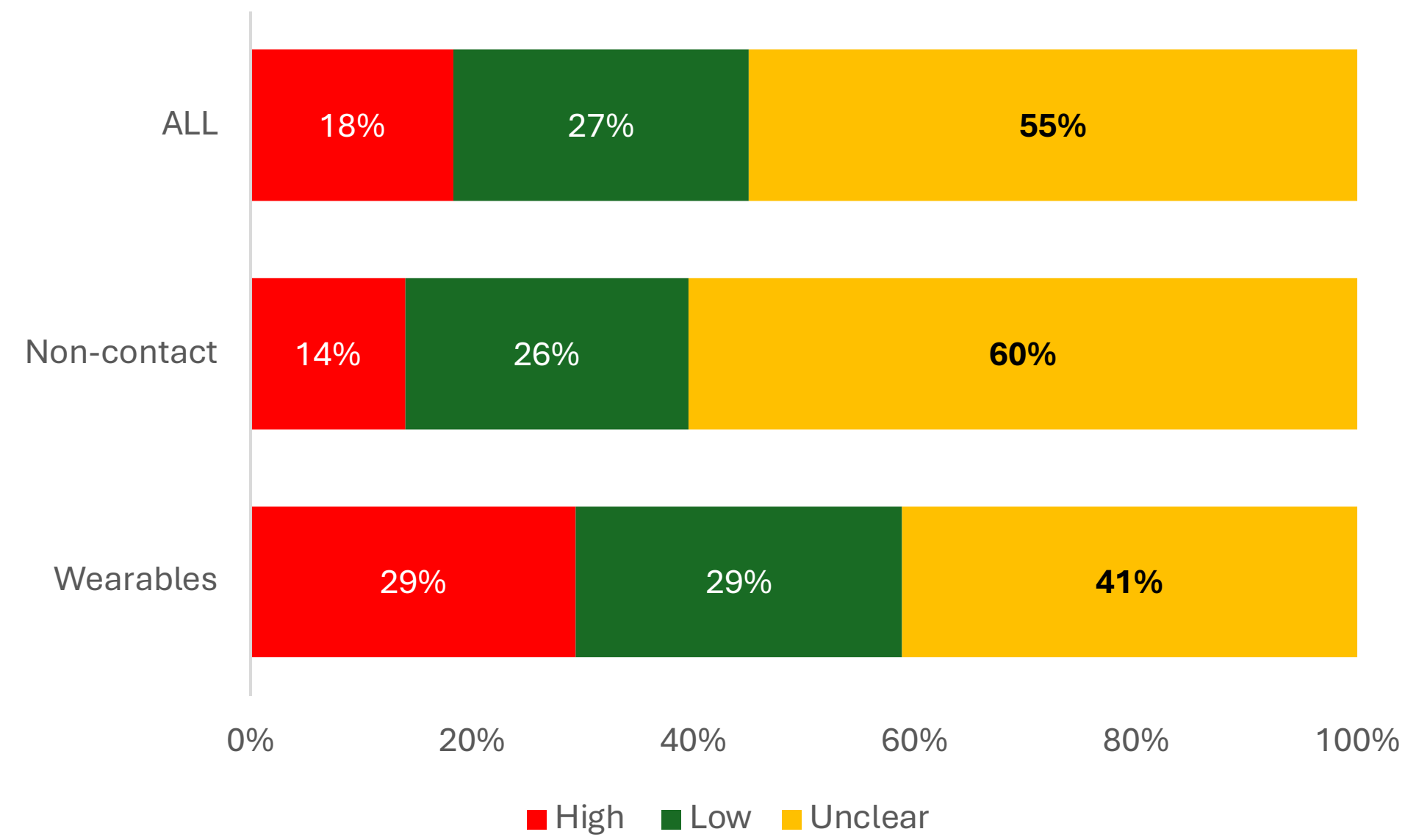

(C) Reference Measurement – Risk of Bias

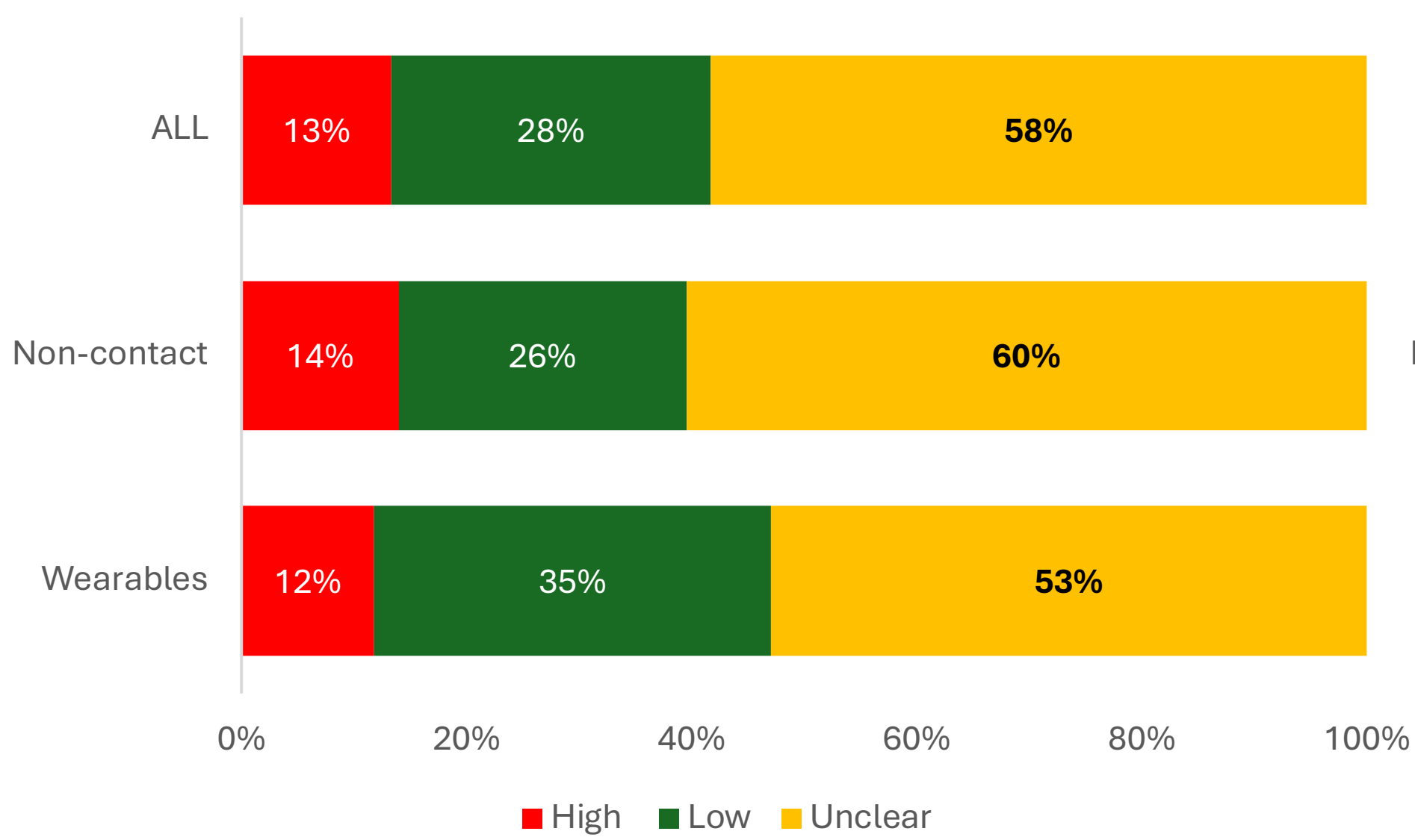

(D) Reference Measurement - Applicability Concerns

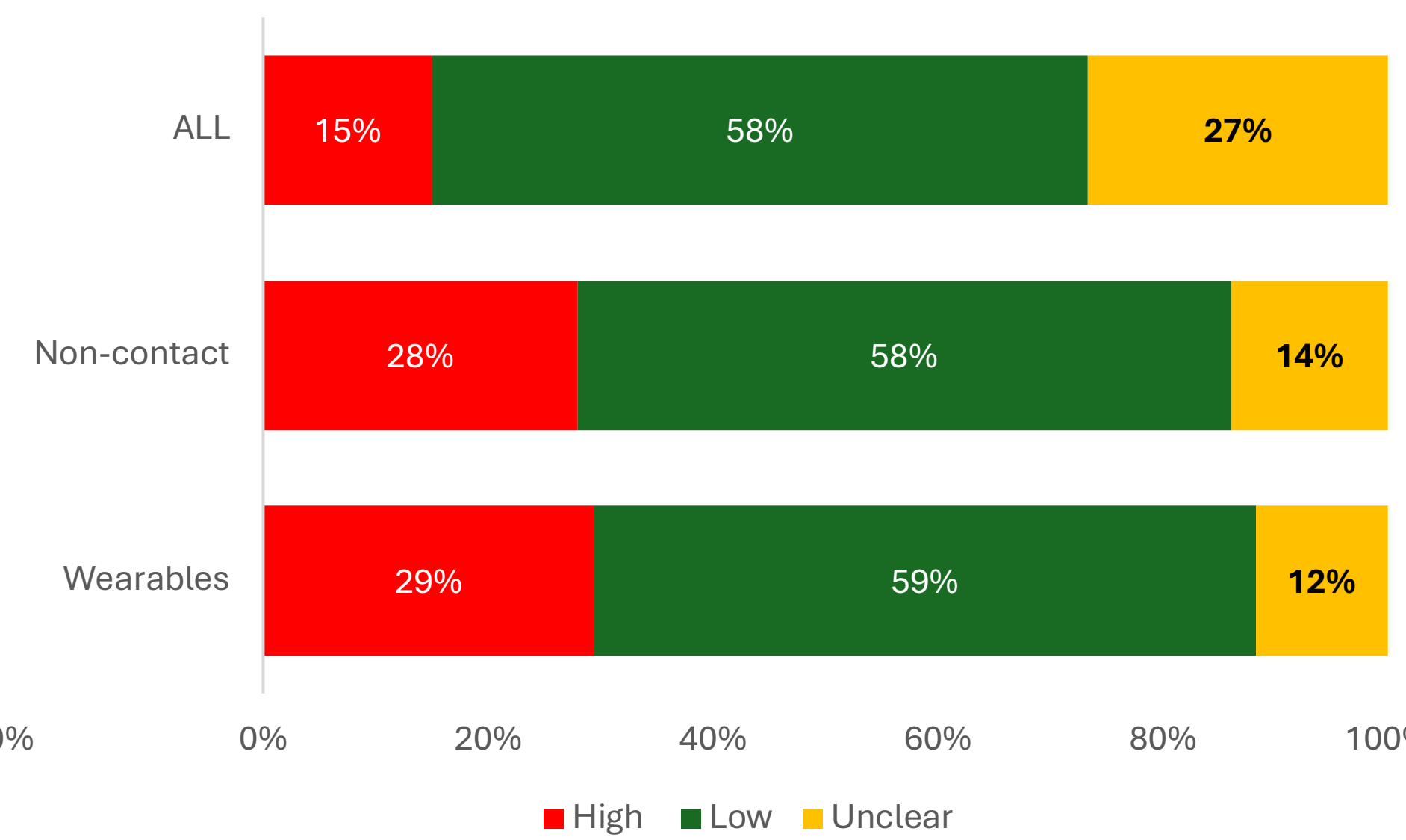

(E) Index Test – Risk of Bias

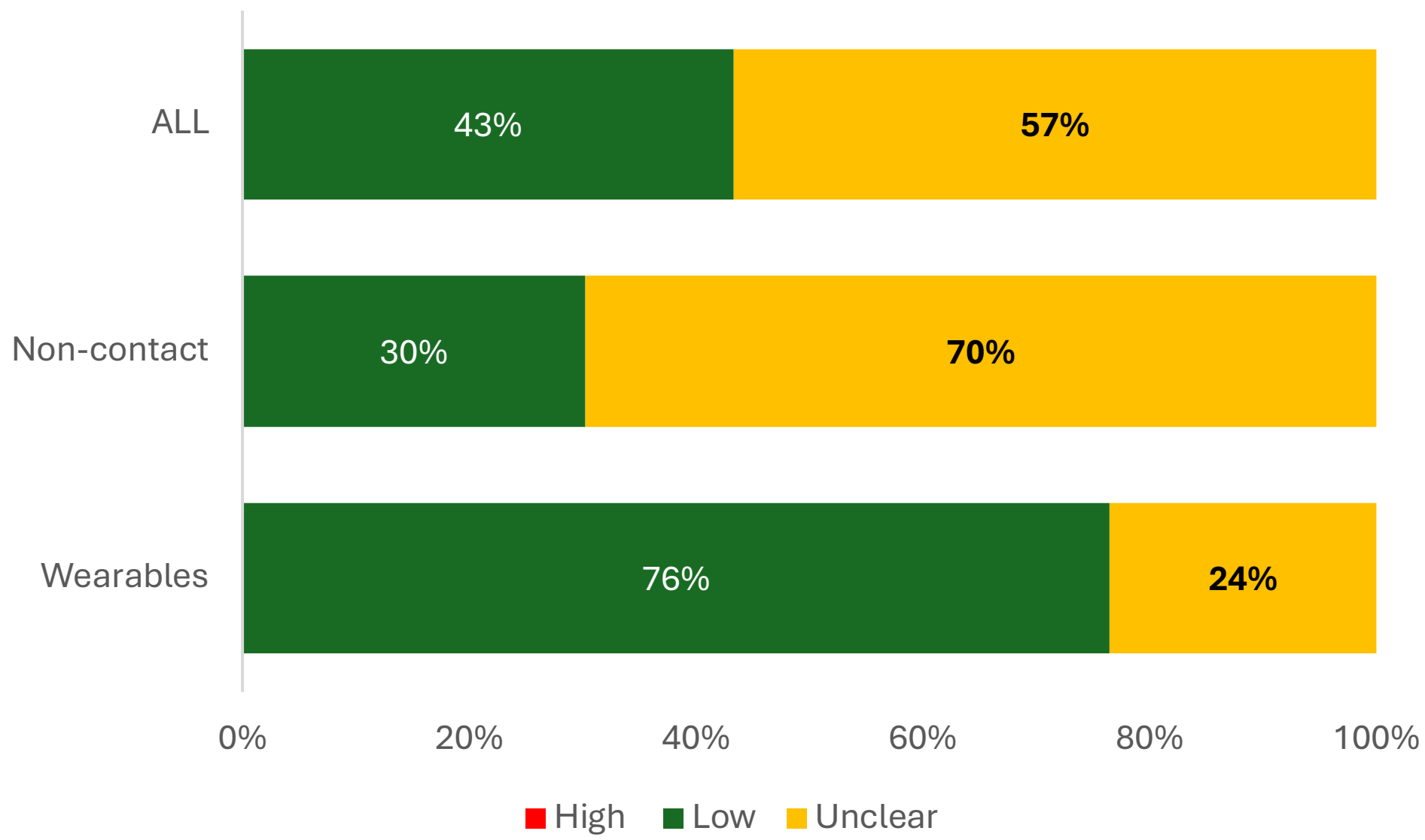

(F) Index - Applicability Concerns

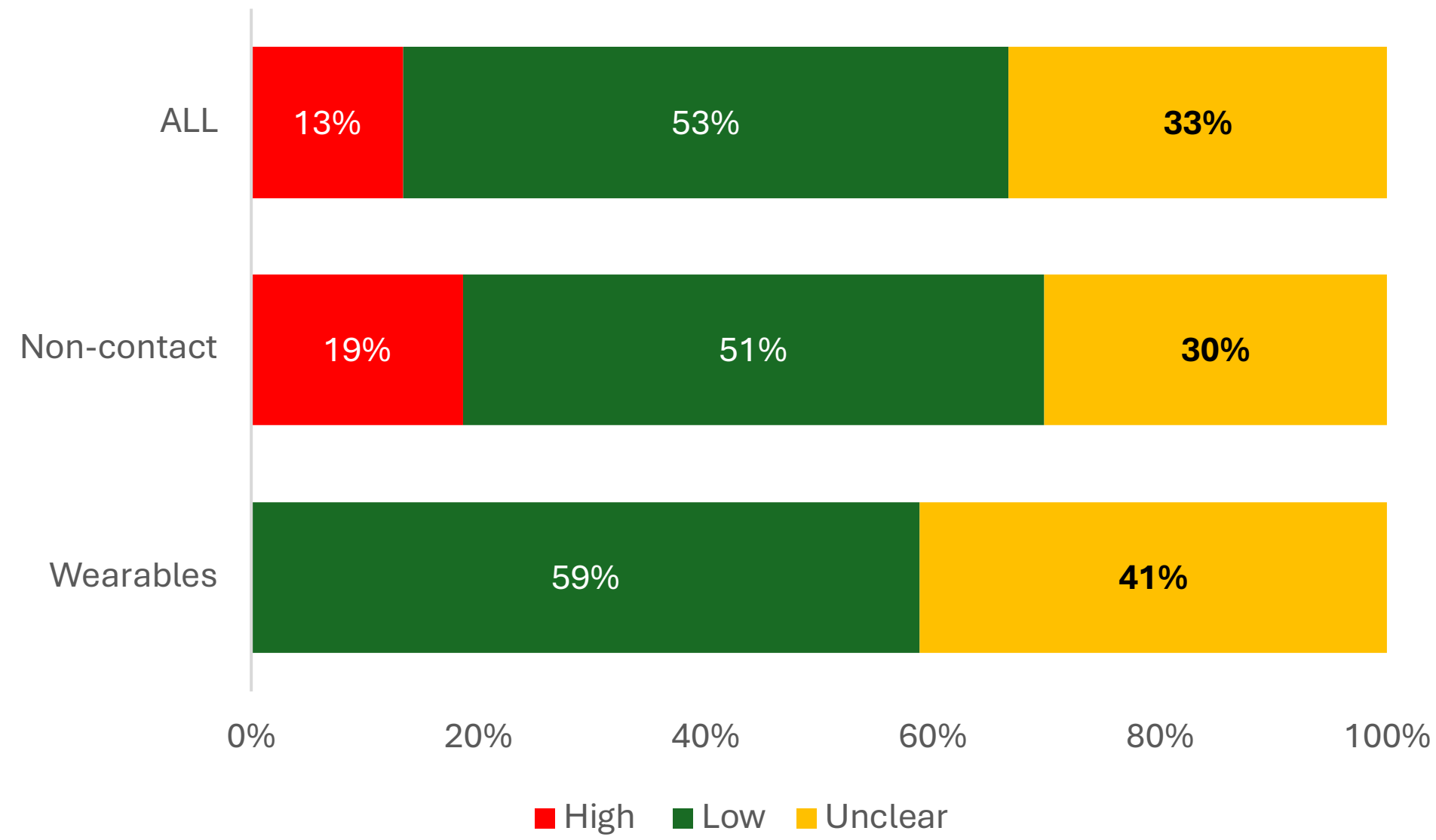

(G) Flow and Timing – Risk of Bias

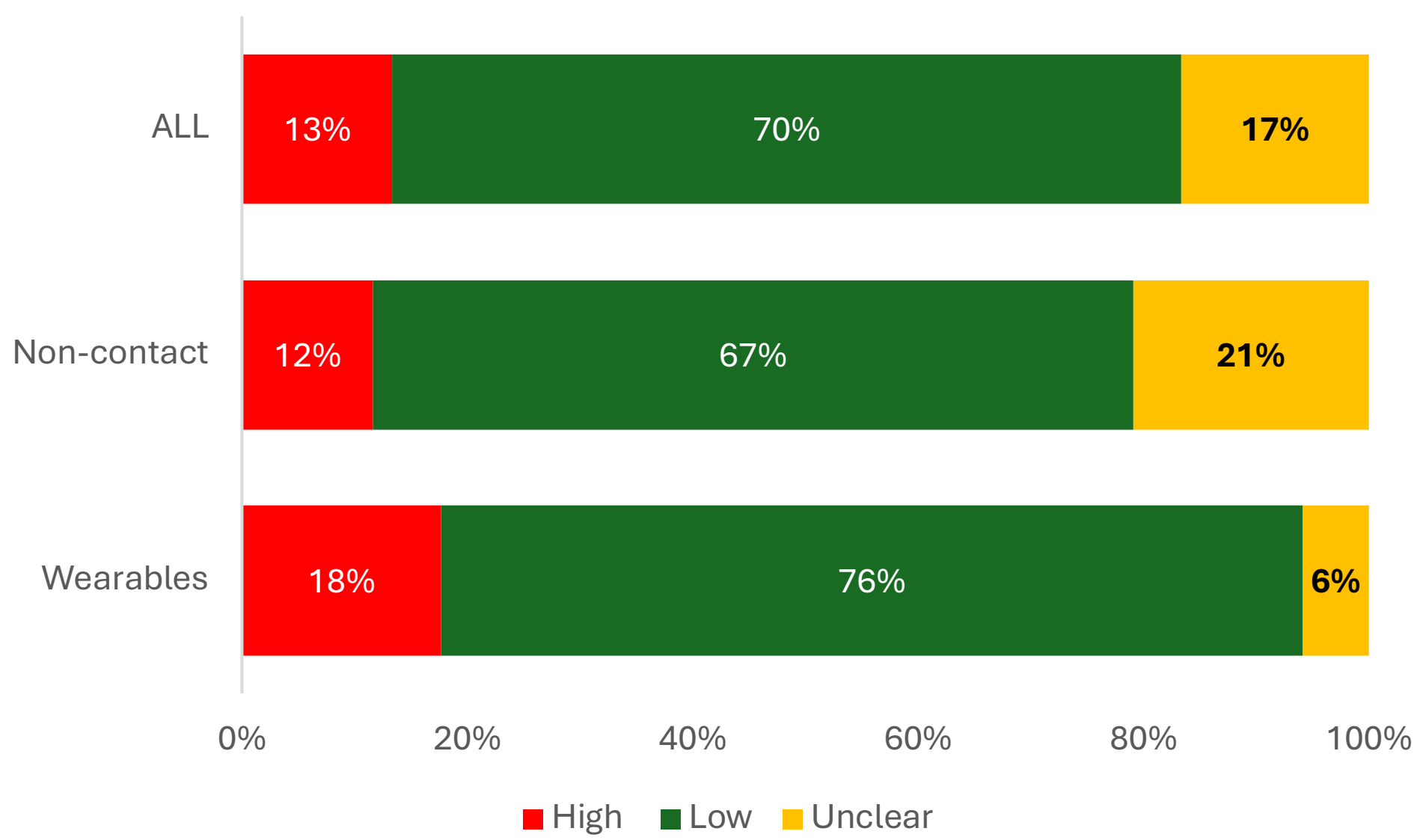

Legend. Results are presented as (%), for all studies (n=60); and non-contact (n=48) and wearables (n=17). Panels A-D represent the risk of bias assessment. Panels E-G represent applicability concerns assessment.

Supplementary Table S4 – Non-Contact Methods by Vital Sign Monitored

**Type of noncontact sensor used for RR**	**31(100)**
RGB Camera	23 (74)
Infrared thermography	8 (26)
Depth Camera	5 (16)
Monochrome camera	3 (10)
Radar	2 (7)
**ROI for RR**	
torso	18 (58)
head	12 (39)
variable	8 (26)
upper limbs	1 (3)
lower limbs	1 (3)
neck	1 (3)
no info	2 (6)
**Type of noncontact sensor used for HR**	**22 (100)**
RGB Camera	20 (91)
Infrared thermography	4 (18)
Monochrome camera	2 (9)
Radar	2 (9)
**ROI for HR**	
torso	13 (59)
head	13 (59)
variable	4 (18)
neck	2 (9)
upper limb	2 (9)
lower limb	1 (5)
no info	1 (5)
**Type of noncontact sensor used for SpO2**	**1 (100)**
RGB Camera	1 (100)
**ROI for SpO2**	**1 (100)**
Head	1 (100)

Legend: results are presented as n (%)

Supplementary Table S5 – Wearable Methods by Vital Sign Monitored

**Sensing method for HR**	**13 (100)**
ECG	7 (54)
PPG	4 (31)
Acoustics	2 (15)
**Sensing method for RR**	**8 (100)**
Impedance	1 (13)
Tensile strength	2 (25)
Acoustics	2 (25)
Accelerometry	2 (25)
DEMG	1 (13)
ECG	1 (13)
**Sensing method for SpO2**	**5 (100)**
PPG	5 (100)
**Sensing method for temperature**	**3 (100)**
Thermistor	2 (67)
Not specified	1 (33)
**Sensing method for blood pressure**	**1 (100)**
Capacitive sensor	1 (100)

Legend: results are presented as n (%)

Supplementary Table S6 HR & RR Accuracy Results

HR Accuracy Results							
	**All**	**Noncontact**	**Wearables**	**p-value**	**Including all data**	**Excluding data**	**p-value**
**Bland Altman**	0.07 (-0.51 to 0.44)	0.28 (-0.22 to 0.76)	-0.17 (-0.95 to 0.02)	0.091	-0.17 (-1.40 to 0.47)	0.25 (-0.23 to-0.43)	0.536
**Lower 95% LoA**	-6.60 (-10.2 to -4.85)	-6.6 (-10.6 to -4.15)	-5.63 (-6.4 to -5.60)	0.932	-7.55 (-9.42 to -5.87)	-5.6 (-11.48 to -4.40)	0.323
**Upper 95% LoA**	5.54 (4.64 to 11.3)	6.79 (4.46 to 12.0)	5 (4.9 to 5.28)	0.485	6.04 (4.43 to 10.7)	5.0 (4.77 to 11)	0.792
**MAE**	3.03 (2.78 to 4.5)	2.96 (2.62 to 4.73)	4.5*	N/A	4.83 (3.59 to 6.20)	2.87 (2.10 to 4.40)	N/A
**RMSE**	3.61 (2.44 to 5.92)	3.78 (2.33 to 6.44)	2.8*	N/A	4.9 (3.56 to 9.57)	2.73 (1.94 to 3.70)	N/A
**RR Accuracy Results**							
	**All**	**Noncontact**	**Wearables**	**p-value**	**Including all data**	**Excluding data**	**p-value**
**Bland Altman**	0.40 (0.18 – 0.80)	0.28 (0.18 to 0.76)	0.76 (0.21 to 0.82)	0.497	0.68 (-0.49 to 0.77)	0.27 (0.17 – 01.08)	N/A
**Lower 95% LoA**	-9.16 (-12.75 to –5.85	-9.15 (-12.6 to -6.50)	-7.62 (-13.1 to -3.17)	N/A	-10.6 (-14.5 to -7.80)	-7.96 (-13.0 to -5.69)	N/A
**Upper 95% LoA**	10.3 (5.58 to 13.5)	10.34 (6.0 to 12.01)	11.62 (4.86 to 18.2)	N/A	7.80 (4.96 to 13.7)	9.40 (5.95 to 14.0)	N/A
**MAE**	3.76 (2.45 – 5.08)	3.56 (2.37 to 4.81)	9.0*	N/A	4.26 (4.04 to 4.49)	3.36 (2.45 to 5.36)	N/A
**RMSE**	5.84 (3.85 – 6.96)	5.84 (3.85 to 6.96)	N/A	N/A	6.8 (5.32 to 7.60)	4.15 (2.90 to 7.42)	0.101

Legend: N/A written in cases where one group had <5 observations and this it was not appropriate to conduct Mann Whitney test. *Indicates only one study reported this outcome and value reported here but no IQR provided for one value. Limits of agreement (LoA), mean absolute error (MAE); root mean square error (RMSE)

Supplementary Table S7 – Key Statements

Included Articles	All	Noncontact	Wearables	p-value
**Funding Statement**	**60 (100)**	**43 (100)**	**17 (100)**	**0.073**
Yes	43 (72)	28 (65)	15 (88)	
No	17 (28)	15 (35)	2 (12)	
**Conflict of interest**				**0.121**
No statement	27 (45)	22 (51)	5 (29)	
No	26 (43)	18 (42)	8 (47)	
Yes	7 (12)	3 (7)	4 (24)	
**Did analysis include all data**				**0.859**
No	28 (47)	21 (48)	7 (41)	
Yes	26 (43)	18 (41)	8 (47)	
Unsure	6 (10)	4 (9)	2 (12)	

Legend: results are presented as n (%)

Supplementary Table S8 – QUADAS 2 Assessment

Included Articles	All (n=60)	Noncontact (n=43)	Wearables (n=17)	p-value
**Participant Selection Risk of Bias**				
High	5 (8)	3 (7)	2 (12)	0.137
Low	19 (32)	11 (26)	8 (47)	
Unclear	36 (60)	29 (67)	7 (41)	
**Concerns Participant Selection Applicability**				
High	11 (18)	6 (14)	5 (29)	0.290
Low	16 (27)	11 (26)	5 (29)	
Unclear	33 (55)	26 (60)	7 (41)	
**Reference Measure Risk of Bias**				
High	8 (13)	6 (14)	2 (12)	0.850
Low	17 (28)	11 (26)	6 (35)	
Unclear	35 (58)	26 (60)	9 (53)	
**Concerns Reference Measure Applicability**				
High	9 (15)	12 (28)	5 (29)	1.00
Low	35 (58)	25 (58)	10 (59)	
Unclear	16 (27)	6 (14)	2 (12)	
**Index Measure Risk of Bias**				
High	0 (0)	0 (0)	0 (0)	< 0.01
Low	26 (43)	13 (30)	13 (76)	
Unclear	34 (57)	30 (70)	4 (24)	
**Concerns Index Measure Applicability**				
High	8 (13)	8 (19)	0 (0)	0.174
Low	32 (53)	22 (51)	10 (59)	
Unclear	20 (33)	13 (30)	7 (41)	
**Flow and Timing Risk of Bias**				
High	8 (13)	5 (12)	3 (18)	0.367
Low	42 (70)	29 (67)	13 (76)	
Unclear	10 (17)	9 (21)	1 (6)	

Legend: results are presented as n (%)

Table E9 – Figures and tables corresponding to each objective

**Objective**	**Figure/Table**
Outline non-contact and wireless vital sign monitoring technologies that have been developed and tested in NICU	Table 1<br>Table 2<br>Table 3<br>Table E4<br>Table E5
Investigate device feasibility, safety, and accuracy	Table 5<br>Table 6<br>Table E6
Examine methodological quality	Figure 3<br>Table E7<br>Table E8
